# Evaluation of Anterior Chamber Volume in Cataract Patients with Swept-Source Optical Coherence Tomography

**DOI:** 10.1155/2016/8656301

**Published:** 2016-09-05

**Authors:** Wenwen He, Xiangjia Zhu, Don Wolff, Zhennan Zhao, Xinghuai Sun, Yi Lu

**Affiliations:** ^1^Department of Ophthalmology, Eye and Ear, Nose, and Throat Hospital, Fudan University, 83 Fenyang Road, Shanghai 200031, China; ^2^Key Laboratory of Myopia, Ministry of Health, Shanghai 200031, China; ^3^Shanghai Key Laboratory of Visual Impairment and Restoration, Fudan University, Shanghai 200031, China; ^4^Parkway Health Hongqiao Medical Center, 2258 Hongqiao Road, Shanghai 200335, China

## Abstract

*Purpose.* To evaluate the anterior chamber volume in cataract patients with Swept-Source Optical Coherence Tomography (SS-OCT) and its influencing factors.* Methods.* Anterior chamber volume of 92 cataract patients was evaluated with SS-OCT in this cross-sectional study. Univariate analyses and multiple linear regression were used to investigate gender, age, operated eye, posterior vitreous detachment, lens opacity grading, and axial length (AXL) related variables capable of influencing the ACV.* Results.* The average ACV was 139.80 ± 38.21 mm^3^ (range 59.41 to 254.09 mm^3^). The average ACV was significantly larger in male patients than in female patients (*P* = 0.001). ACV was negatively correlated with age and LOCS III cortical (C) grading of the lens (Pearson's correlation analysis, *r* = −0.443, *P* < 0.001, and Spearman's correlation analysis, *ρ* = −0.450, *P* < 0.001). ACV was also increased with AXL (Pearson's correlation analysis, *r* = 0.552, *P* < 0.001). Multiple linear regression showed that, with all of the covariates entered into the model, gender (*P* = 0.002), age (*P* = 0.015), LOCS III C grade (*P* = 0.043), and AXL (*P* = 0.001) were still associated with ACV (*F* = 10.252  *P* < 0.001  *R* ^2^ = 0.498).* Conclusion.* With SS-OCT, we found that, in healthy cataract patients, ACV varied significantly among different subjects. Influencing factors that contribute to reduced ACV were female gender, increased age, LOCS III C grade, and shorter AXL.

## 1. Introduction

Precise measurement of the anterior chamber is important in many aspects of ophthalmology, such as assessing glaucoma risk [1, 2] and surgical planning and intraocular lens (IOL) power calculation [3, 4]. Previous studies have focused on the measurement of anterior chamber depth (ACD) or the anterior chamber angle (ACA). Accurate measurement of anterior chamber volume (ACV) has been difficult historically due to technology limitations.

Recent development of Swept-Source Optical Coherence Tomography (SS-OCT CASIA SS-1000 OCT, Tomey Corporation, Nagoya, Japan) is a form of Fourier-domain OCT (FD-OCT), which uses a monochromatic tunable fast scanning laser source and a photodetector to detect wavelength-resolved interference signal instead of using a spectrometer as in spectral-domain OCT (SD-OCT) [5]. Thus, with 30,000 A-scans per second, SS-OCT allows rapid and precise anterior chamber measurement. As a variation of FD-OCT, SS-OCT has advantages of measurement speed and sensitivity over time-domain OCT [6]. Besides, SS-OCT also has advantages over SD-OCT, such as higher robustness [7] and capability of separating the real OCT image from its mirror image [8, 9]. Previous studies comparing SS-OCT and SD-OCT in posterior segment measurements have proved these advantages. It has been shown to have higher resolution, resulting in more measureable images [10]. SS-OCT is also superior in detecting details of choroid-scleral interface and choroidal sublayer [11, 12]. Studies comparing SS-OCT to other OCTs in observations of anterior segment were rare, yet SS-OCT has been widely used in the measurement of anterior chamber [13–16].

Anterior chamber anatomy may vary with aging, thickening of the lens, liquefaction of the vitreous, corneal changes, gender differences [17], and elongation of the axial length (AXL) in high myopia [18, 19]. Cataract types also have different impact on anterior chamber characteristics [20]. However, few studies have evaluated the effects of all these factors on the volume of the anterior chamber in normal cataract patients. In this study, we used the SS-OCT, to investigate ACV in normal cataract patients and to better understand factors that impact ACV measurement.

## 2. Methods

The Institutional Review Board of the Eye and ENT Hospital of Fudan University approved this prospective study. All procedures adhered to the tenets of the* Declaration of Helsinki* and were conducted in accordance with the approved research protocol. Informed consent was obtained from each patient. The study was registered at https://www.clinicaltrials.gov/; the clinical trial accession number is NCT02182921.

### 2.1. Subjects

Ninety-two eyes of consecutive cataract patients at the Eye and ENT Hospital of Fudan University, between May 2015 and August 2015, were enrolled. Exclusion criteria included zonular weakness, corneal disease, glaucoma, previous trauma, or any ocular surgical history.

### 2.2. Examinations

Complete anterior and dilated funduscopic exams were done. Posterior vitreous detachment (PVD) was evaluated by B-scan by the same senior technician. AXL was measured with an IOL Master (Carl Zeiss AG, Oberkochen, Germany). The lens opacity was assessed according to the Lens Opacities Classification System III (LOCS III developed by Dr. Leo Chylack, Mass Eye and Ear) under slit lamp examination by one investigator. LOCS III System has 4 categories: NC = nuclear color, NO = nuclear opalescence, C = cortical cataract, and P = posterior subcapsular cataract. According to the previous study, NC and NO were graded from 0.1 to 6.9 and C was graded from 0.1 to 5.9 [20]. P was graded from 1 to 5.

**Figure 1:**
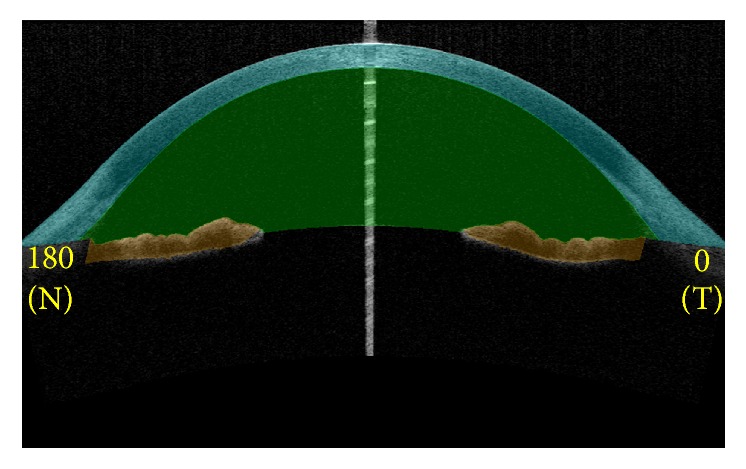
SS-OCT automatically detects the boundaries of cornea, iris and lens. SS-OCT= Swept-Source Optical Coherence Tomography.

SS-OCT anterior segment scan mode was used with 128 radial scans, with a depth of 6 mm and a length of 16 mm. Patients were instructed to fixate on the internal target and pull down the lower lid while the technician elevated the upper lid to expose the limbus. The total scan time was less than 0.3 seconds. All images with eyelids or motion artifact were excluded. The corneal map scan type was used for measurement of posterior corneal curvature. All eyes were imaged in room light (336 lux) without pupil dilation. A total of 64 B-scans taken from the anterior segment scan mode were analyzed for measurement of ACV. The instrument software automatically detected the boundaries of cornea, iris, and lens for each image, as shown in Figure 1. Manual adjustment was made if the software failed to detect the boundaries at the correct location. Angle width parameters derived from SS-OCT included the angle opening distance 500 (AOD 500), the trabecular iris space area 500 (TISA 500), the angle recess area 500 (ARA 500), and the trabecular iris angle 500 (TIA 500), which were determined as previously described for the superior, inferior, nasal, and temporal angles [21].

### 2.3. Statistical Analysis

All data were expressed as the mean ± standard deviation. Student's *t*-test was used to compare differences in mean measurements between men and woman after normality tests and homogeneity of variance tests. Pearson and Spearman's correlation analyses were used to investigate the relationships between ACV and demographic data (Pearson for continuous variables and Spearman for categorical variables). Determinants of ACV were further evaluated using multiple liner regression. *P* values < 0.05 were considered statistically significant. All analyses were performed using SPSS version 11.0 (SPSS Inc., Chicago, IL, USA).

## 3. Results

### 3.1. Patient Characteristics

The demographic data of the patients are shown in Table 1. The average age of the patients was 66.48 ± 10.57 years old. There were 29.35% (27/92) high myopic (<−6.0 D) patients and the average AXL was 25.09 ± 2.96 mm.

### 3.2. Biometry Measurements of ACV Related Anterior Segment Parameters

Table 2 shows the biometry measurements of anterior segment. The average ACV was 139.80 ± 38.21 mm^3^, range 59.41 to 254.09 mm^3^. The average ACD was 2.71 ± 0.42 mm.

### 3.3. Influencing Factors of ACV

**Figure 2:**
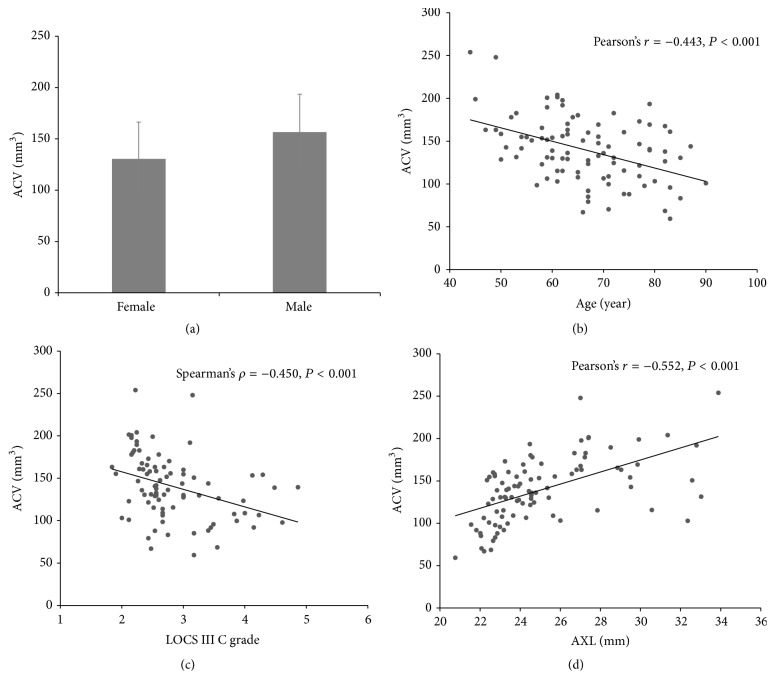
Univariant analysis. (a) The average ACV was significantly greater in males compared to females (*P* = 0.001). (b) ACV was negatively correlated with age (Pearson's correlation analysis, *r* = −0.443, *P* < 0.001). (c) ACV was negatively correlated with LOCS III C grade of the lens (Spearman's correlation analysis, *ρ* = −0.450, *P* < 0.001). (d) ACV was also increased with AXL (Pearson's correlation analysis, *r* = 0.552, *P* < 0.001). ACV = anterior chamber volume; AXL = axial length. LOCS III = Lens Opacities Classification System III; C = cortical opacity.

We performed univariate analysis for influencing factors of ACV and multivariable analysis to adjust for independent covariates. The average ACV was significantly larger in male patients than in female patients (Figure 2(a), *P* = 0.001). ACV was negatively correlated with age and LOCS III C grade of the lens (Figures 2(b) and 2(c), Pearson's correlation analysis, *r* = −0.443, *P* < 0.001, and Spearman's correlation analysis, *ρ* = −0.450, *P* < 0.001). LOCS III NC, NO, and P grades were not correlated with ACV narrowing (Spearman's correlation analysis, *ρ* = 0.127, *P* = 0.246, *ρ* = 0.146, *P* = 0.180, *ρ* = −0.024, and *P* = 0.819, resp.). ACV was also increased with increased AXL (Figure 2(d), Pearson's correlation analysis, *r* = 0.552, *P* < 0.001).

Table 3 presents the result from the multiple linear regression. With all of the covariates entered into the model, female gender (*P* = 0.002), greater age (*P* = 0.015), higher LOCS III C grade (*P* = 0.043), and less myopia AXL (*P* = 0.001) were all correlated with decreased ACV (*F* = 10.252  *P* < 0.001  *R* ^2^ = 0.498).

## 4. Discussion

Objective, precise measurement of the anterior chamber volume has importantsignificance as a predictor of narrow angle glaucoma risk, assessment of pupil block, in addition to surgical planning in AC IOL and Phakic IOL placement. Previous reports of anterior chamber volume measurements relied on the Scheimpflug system [22], which requires protracted cooperation of patients during testing. By using Scheimpflug system, previous studies found that, with increasing age, ACD and ACV diminished and no correlation was found between ACV and anterior chamber angle [22]. However, the Scheimpflug system could only provide an estimation rather than a direct visualization of the anterior chamber [23], resulting in an inaccurate assessment of ACV which also lacks reproducibility. CASIA SS-1000 OCT used a swept laser source, which could show high-resolution 3-dimensional images of anterior chamber by a very high-speed scanning system. Previous studies reported variable influencing factors on ACV, including gender [17], ACD, and age [20]. However, it remains unclear whether these factors remain significant after adjusting for independent covariates. In the study, we reported SS-OCT data in healthy cataract patients and found that ACV varied significantly among different subjects. Influencing factors that contributed to reduced ACV were female gender, increased age, LOCS III C grade, and lower AXL.

The female gender was related to decreased ACV possibly because generally females have shorter AXL, smaller body habitus, eyes, and therefore a narrower anterior chamber than males [17]. However, there were almost twice as many females as males in our study, which might be a bias. This was a limitation and we will verify the result in a population with similar percentages of men and women in the future. Besides, the anterior chamber became shallower over time with thickening of the crystalline lens, which partly explained the negative correlation between increased age and ACV. Cortical lens changes impact lens thickness more significantly than nuclear sclerosis. Consequently, LOCS III C grade was also negatively correlated with ACV. After adjusting for independent covariates, gender, age, LOCS III C grade, and AXL were still related to the volume of anterior chamber by multiple linear regression analysis.

These factors, along with SS-OCT evaluation, are important for assessing risk in glaucoma patients and may impact the timing of therapeutic cataract surgery in patients with anatomic narrow angles or those with cataract-induced narrow angles. Moreover, patients needing AC IOL or Phakic IOL placement can benefit from SS-OCT assessment as a preoperative tool to assess risk and aid surgical planning. Further studies can examine if these risk factors are also found in patients before senile cataracts develop, in patients with open or narrow angle glaucoma, and how cataract surgery impacts the postoperative ACV in patients at risk of narrow angles.

In addition, several anterior segment imaging modalities, such as Scheimpflug system, anterior segment OCT, and ultrasound biomicroscopy (UBM), can be used to evaluate the volume of the anterior chamber. All devices promise quantitative information and qualitative imaging of anterior segment. However, compared to other instruments, the SS-OCT used in the current study has several advantages: (1) It is a noncontact optical system compared to UBM, which avoids distortion of the eye anatomy and angle and reduces contagion [24]. (2) It is faster than the Scheimpflug system or UBM because of the swept laser source and captures data of ACV in less than 1 second. Thus, patients could cooperate with this test easier than the Scheimpflug system or UBM. (3) Similarly to UBM, SS-OCT provides direct angle visualization and generates an ACV which is more precise and objective than the Scheimpflug system of angle estimation. Besides, this SS-OCT could automatically show accurate measurements of anterior chamber angle, which has advantages over UBM [14]. However, other methods still have their value in clinical ophthalmology. For instance, in cases of corneal scaring, UBM could still evaluate anterior chamber parameters while OCTs could not.

To conclude, SS-OCT could provide faster, objective, and more precise measurement of anterior segment than other methods in some conditions, which may have advantages in evaluation of ACV but still need further confirmation in the future study. With SS-OCT, we found female gender, increased age, higher LOCS III cortical grading, and decreased AXL to be important predictors for smaller ACV in normal cataract patients.

## Figures and Tables

**Table 1:** Demographic data.

Parameter	Data (range)
Age (year)	66.48 ± 10.57 (44–90)
Gender (male/female)	33/59
Operated eye (right/left)	43/49
AXL (mm)	25.09 ± 2.96 (20.76–33.88)
High myopia (< −6.00) (%)	29.35 (27/92)
LOCS III NC grade	3.27 ± 0.97 (1.8–5.7)
LOCS III NO grade	3.14 ± 0.94 (1.5–5.5)
LOCS III C grade	2.83 ± 0.67 (1.8–4.9)
LOCS III P grade	1.41 ± 0.58 (1.0–3.0)

Values are presented as the mean ± standard deviation (range) or *n*.

AXL = axial length; LOCS III = Lens Opacities Classification System III; NC = nuclear color; NO = nuclear opalescence; C = cortical cataract; P = posterior subcapsular cataract.

**Table 2:** Biometry measurements of anterior segment.

Parameter	Data (range)
ACV (mm^3^)	139.80 ± 38.21 (59.41–254.09)
ACD (mm)	2.71 ± 0.42 (1.76–3.86)
AOD 500 (mm)	0.61 ± 0.28 (0.19–1.67)
ARA 500 (mm^2^)	0.35 ± 0.18 (0.11–1.25)
TISA 500 (mm^2^)	0.24 ± 0.12 (0.07–0.74)
TIA 500 (°)	32.72 ± 11.62 (9.53–61.55)
Posterior corneal curvature (D)	6.40 ± 0.26 (−5.80–−6.95)

Values are presented as the mean ± standard deviation (range).

ACV = anterior chamber volume; ACD = anterior chamber depth; AOD 500 = angle opening distance 500; ARA 500 = angle recess area 500; TISA 500 = trabecular iris space area 500; TIA 500 = trabecular iris angle 500.

**Table 3:** Multiple linear regression analysis of ACV.

	*β*	*P*
Gender	0.301	0.002^*∗*^
Age	−0.263	0.015^*∗*^
Operated eye	−0.087	0.325
PVD	0.053	0.597
LOCS III NC grade	−0.254	0.393
LOCS III NO grade	0.208	0.477
LOCS III C grade	−0.183	0.043^*∗*^
LOCS III P grade	−0.029	0.733
AXL	0.358	0.001^*∗*^

Model *F* = 10.252  *P* < 0.001  *R* ^2^ = 0.498.

PVD = posterior vitreous detachment; LOCS III = Lens Opacities Classification System III; NC = nuclear color; NO = nuclear opalescence; C = cortical cataract; P = posterior subcapsular cataract; AXL = axial length.

^*∗*^These parameters were significantly correlated with ACV (*P* < 0.05).
